# High-Performance PMN-PT Single-Crystal-Based 1-3 Composite Transducer Integrated with a Biopsy Needle

**DOI:** 10.3390/bios14020074

**Published:** 2024-01-31

**Authors:** Benjamin C. Kreager, Huaiyu Wu, Wei-Yi Chang, Sunho Moon, Josh Mitchell, Chang Peng, Chih-Chung Huang, Marie Muller, Jian Tian, Xiaoning Jiang

**Affiliations:** 1 Department of Mechanical and Aerospace Engineering, North Carolina State University, Raleigh, NC 27695, USA; bkreage@ncsu.edu (B.C.K.); hwu15@ncsu.edu (H.W.); smoon4@ncsu.edu (S.M.); jmitche2@alumni.ncsu.edu (J.M.); mmuller2@ncsu.edu (M.M.); 2 CTS Advanced Materials, 4925 Indiana Ave, Lisle, IL 604532, USA; wei-yi.chang@ctscorp.com (W.-Y.C.); jian.tian@ctscorp.com (J.T.); 3 School of Biomedical Engineering, ShanghaiTech University, Shanghai 201210, China; pengchang@shanghaitech.edu.cn; 4 Department of Biomedical Engineering, National Cheng Kung University, Tainan 701, Taiwan; cchuang@mail.ncku.edu.tw

**Keywords:** imaging-guided biopsy, high-frequency ultrasound, piezoelectric, fine needle aspiration, ultrasound imaging

## Abstract

To address the need for high-resolution imaging in lung nodule detection and overcome the limitations of the shallow imaging depth associated with high-frequency ultrasound and the complex structure of lung tissue, we successfully integrated 50 MHz ultrasound transducers with 18-gauge biopsy needles. Featuring a miniaturized size of 0.6 × 0.5 × 0.5 mm^3^, the 50 MHz micromachined 1-3 composite transducer was tested to perform mechanical scanning of a nodule within a lung-tissue-mimicking phantom in vitro. The high-frequency transducer demonstrated the ability to achieve imaging with an axial resolution of 30 μm for measuring nodule edges. Moreover, the integrated biopsy needle prototype exhibited high accuracy (1.74% discrepancy) in estimating nodule area compared to actual dimensions in vitro. These results underscore the promising potential of biopsy-needle-integrated transducers in enhancing the accuracy of endoscopic ultrasound-guided fine needle aspiration biopsy (EUS-FNA) for clinical applications.

## 1. Introduction

Cancer is one of the major causes of mortality in the world. In the United States in 2020, more than 1.6 million new cancer diagnoses were reported along with at least 600,000 fatalities [1]. Prompt diagnosis is critical for effective cancer intervention and favorable patient outcomes, with the goal of preventing progression to an advanced cancer stage characterized by the detachment and spread of cancer cells from the original tumor to neighboring organs and tissues, which is known as metastasis [2,3,4,5,6,7]. Thus, early cancer diagnosis methods have been developed to combat potential metastatic cancer, including imaging devices, needle biopsies, blood tests, endoscopic procedures, and genetic testing.

One early diagnosis method that is utilized extensively is fine needle aspiration (FNA) for acquiring samples of suspicious tissues residing within the lung, breast, liver, kidney, thyroid, and bones. Compared with open surgery, fine-needle biopsies are preferred since they are minimally invasive and efficient [8,9]. During a standard FNA procedure, a healthcare professional guides an 18–27-gauge needle to the target tissue with guidance from imaging modalities such as ultrasound (US) imaging, magnetic resonance imaging (MRI), computed tomography (CT), or fluoroscopy, and then a piece of the targeted tissue is suctioned out with a 10 mL syringe attached to the biopsy needle [9,10,11]. While conducting this task, a small needle diameter is critical to minimize surrounding tissue damage and for a more precise sample collection, yet also increases the difficulty of needle guidance clinically [8,12]. Even with the assistance of external imaging guidance, false-negative diagnoses appear with variable frequency depending on the target tissue, technical factors, and expertise of the endosonographer [13,14,15].

Misplacement of the needle during operation may cause unintended damage to neighboring tissues or organs, which also requires multiple operations for the collection of the pathological lesion. With each misplacement, the risk of pneumothorax (partial or full lung collapse) increases [16]. It is estimated that misplacement of the needle during biopsy procedures leads to an additional burden of more than USD 1 billion in healthcare costs every year [16]. To enhance the success rate of the biopsy, researchers have demonstrated the use of sensors integrated into biopsy needles or external devices [8,13,16,17,18,19,20,21,22,23,24]. For these devices, real-time monitoring and high resolution are critical to ensure that the operator is able to guide the needle to the correct target [25,26]. Yet, there are limitations to existing biopsy needle guidance methods. CT-scan-guided needle biopsy of lung nodules has become a well-established diagnostic technique [27]. While it identifies the lesion location with sub-millimeter accuracy, this precision is not successfully leveraged due to non-real-time guidance during the biopsy. It has been reported that lung nodules smaller than 10 mm in diameter cannot be reliably sampled and the overall successful acquisition rate is approximately 77% [28]. MRI guidance, similarly, often lacks real-time imaging capabilities, potentially causing difficulties in tracking respiratory motion and precise needle navigation during the procedure [29]. Additionally, fluoroscopy, while enabling real-time imaging, is not able to image smaller lesions due to low contrast sensitivity between adjacent tissues [30]. Thus, external ultrasound imaging is still considered the gold standard for guiding fine needle aspiration [31] due to its high frame rate, portability, relatively low cost, and non-ionizing characteristics [32]. However, the use of low-frequency (5–15 MHz) arrays is unable to sense micrometastases, which have been documented to be as little as 200 μm in length [33]. Conversely, higher-frequency ultrasound does not have the penetration depth required to visualize deeper internal organs and tissues [13,18,19]. For diagnosis of tumors within the chest, external US guidance may also be affected by acoustic reflections from thoracic bones and air [34,35].

To overcome the limitations regarding external ultrasound, several investigations have incorporated sensors into the tip of the needle to facilitate the differentiation of tissue characteristics. One explored modality involved electrical impedance sensing. However, its capability is limited, providing information solely pertaining to the boundary between the transducer and the adjacent medium, excluding the imaging of deeper tissues [8,17,36]. Optoelectrical sensors and physical/chemical arrays were also explored, but similar to electrical impedance sensing, those sensing methods require sophisticated interpretation algorithms and have a limited sensing scope, except for adjacent tissues [20,24,37].

Meanwhile, for minimally invasive ultrasound detection, several ideas have been examined. Micro-electro-mechanical systems (MEMS) have been reported to produce low-intensity ultrasound waves, which would enhance the positional tracking of the external ultrasound probe. This method improves tracking of the needle position but does not assist with locating the lesion without any imaging enhancement of the lung nodule region [16]. Additionally, some researchers have developed ultrasound arrays to be integrated into a biopsy needle [18,19]. These arrays were not implemented inside biopsy needles due to high numbers of ultrasound elements and space limitations for wire paths in the needles. Researchers have demonstrated the feasibility of using high-frequency ultrasound transducers integrated with needles, but few applications have been applied to FNA due to the high attenuation and limited penetration depth [38,39,40]. However, by using a 1-3 composite transducer, we hypothesize that the increased loop sensitivity will allow for a relatively high signal-to-noise ratio. Additionally, the enhanced bandwidth of 1-3 composite transducers provides high axial resolution, which would demonstrate clearer boundaries between adjacent tissues. A comparison of axial and lateral resolutions of the aforementioned needle-based imaging devices is provided in Table 1 below.

Therefore, to address the need for high-resolution imaging in lung nodule detection and overcome the limitations of the shallow imaging depth associated with high-frequency transducers, we successfully integrated 30 MHz (previously prototyped) [41] and 50 MHz ultrasound transducers with 18-gauge biopsy needles. With micromachined PMN-PT 1-3 composite, a miniaturized transducer (0.6 × 0.5 × 0.5 mm^3^) was designed, fabricated, and characterized. For in vitro testing, the prototyped transducer-integrated needles were controlled by a stepper motor and a 3D motion stage to perform mechanical scanning of a nodule within a phantom for nodule boundary detection.

## 2. Materials and Methods

### 2.1. PMN-PT 1-3 Composite Design and Fabrication

PMN-PT 1-3 composite materials have been preferred in ultrasound transducer development due to their exceptional electromechanical coupling factor, which enhances the sensitivity of ultrasound transducers, and remarkable features of low acoustic impedance (<20 MRayl), which facilitate better impedance matching with biological tissues. Additionally, the PMN-PT 1-3 composite material has a relatively higher coupling coefficient compared with bulk materials to provide a better bandwidth [42,43,44,45]. Thus, in this work, a PMN-PT 1-3 composite was chosen as the active material for the transducer’s development.

**Figure 1:**
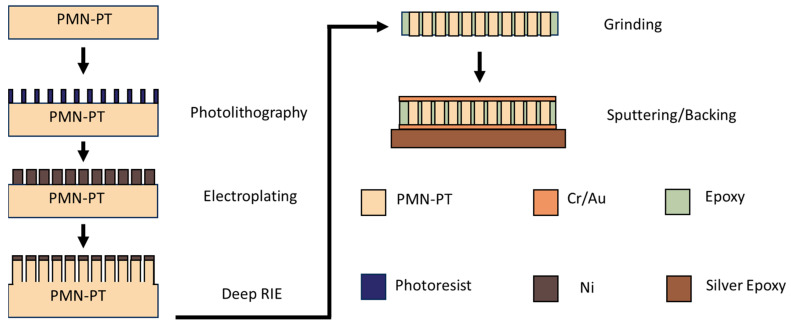
Illustration of the high-frequency composite fabrication process.

To design the 1-3 composite, the most commonly used method is the effective medium model (EMM) [44]. This approach is based on the fundamental assumption that the composite can be regarded as a homogenous medium with adjusted material parameters, providing that the dimensions of the constituent pillars and the kerf spacing are significantly finer than all relevant acoustic wavelengths. With EMM, the effective 1-3 composite properties were derived with active piezoelectric material (PMN-28PT, CTS Corporation, Bolingbrook, IL, USA) and polymer matrix of epoxy (EPO-TEK 301, Epoxy tech. Inc., Billerica, MA, USA). Due to the high operation frequency of the transducer, the PMN-PT crystal 1-3 composite was prepared using the deep reactive ion etching method (CTS corporation Bolingbrook, IL, USA), with which the kerf size was precisely controlled [42]. As shown in Figure 1, a Cr/Au-coated PMN-PT crystal plate was patterned with KMPR photoresist (MicroChem, Westborough, MA, USA) using photolithography as a template for the subsequent nickel electroplating. The thick nickel mask was then electroplated through a circular or square-shaped photoresist template [46]. The photoresist was then removed by an oxygen plasma-etching process (March PM-600 Plasma Asher, Nordson March, Concord, CA, USA). With the hard nickel mask left on the crystal plate, deep reactive-ion etching (DRIE) was performed to etch the crystal where the part was not covered by Ni, forming crystal pillars and kerfs [47]. The etched crystal plate was then filled with an epoxy resin (EPO-TEK 301, Epoxy Technology Inc. Billerica, MA, USA). Precision lapping was processed to achieve the desired thickness after the epoxy was cured. Then, two sides of the composite were deposited with Cr/Au as electrodes. The composite was then poled with a DC electric field of 10 kV/cm for 10 min at room temperature.

### 2.2. 50 MHz 1-3 Composite Transducer Design and Fabrication

The design of the transducer was analyzed using the KLM (Krimholtz, Leedom, and Mattaei) model [48]. Based on the simulation, the aperture size was set as 0.5 × 0.6 mm^2^ to meet the electrical impedance matching of the driving system. The silver epoxy (E-solder 3022, Von-Roll Inc., Cleveland, OH, USA) was chosen as the backing material due to its relatively high acoustic impedance and attenuation ratio. The parylene C layer was applied as the matching layer, the thickness of which can be precisely controlled. The thickness, aperture, and material of each part were determined comprehensively. The design parameters are summarized in Table 2.

For the 50 MHz transducer fabrication, the conductive backing material was cast onto a free-standing 1-3 composite material which was lapped to the final thickness (37 μm). The conductive epoxy was then cured for 24 h at room temperature. The backing layer was then lapped down to a thickness of approximately 500 μm. The piezo and backing stack were subjected to a dicing process to achieve the designed aperture size (DAD 320, DISCO Corporation, Tokyo, Japan). Then, a 24-gauge coaxial cable was connected to the top and bottom of the transducer as the electrodes with the silver epoxy and cured at room temperature for 24 h. The prototyped transducers were coated with a layer of parylene C with a thickness of 9 μm as the matching and passivation layer. Subsequently, each transducer was mounted on the tip of an 18-gauge needle with epoxy (EPO-TEK 301, Epoxy tech, Inc., Billerica, MA, USA).

### 2.3. Transducer Characterization

The prototyped transducer was characterized by measuring the electric impedance and phase spectrum of a pulse/echo response. To measure the electric impedance, the transducer was connected to an impedance analyzer (4294A, Agilent Tech. Inc., Santa Clara, CA, USA), and the impedance and phase were measured at a frequency range of 20–80 MHz. A pulser/receiver (5900 PR, Olympus, Center Valley, PA, USA) was used to excite the transducer with a PRF of 200 Hz and pulse energy of 2 μJ with a gain of 14 dB. A steel bar was used as the reflector at a distance of 2 mm. The RF signal was collected with an oscilloscope (DSO7104B, Agilent Technologies, Santa Clara, CA, USA). The loop sensitivity and the bandwidth of the prototyped transducers were then evaluated based on the measured pulse/echo response. The loop sensitivity [49] is calculated as

$$Loop\;Sensitivity\;\left(dB\right)=20\times \log\left(\frac{V_{out}}{V_{in}}\right)$$

where *V_out_* is the received voltage from the echo signal and *V_in_* is the input signal to the ultrasound transducer. Meanwhile, the bandwidth [50] is computed as

$$\left(-6\;db\right)\;Bandwidth\;\left(\%\right)=\frac{f_{high}-f_{low}}{f_{center}}\times 100\%$$

where *f_high_* and *f_low_* are the upper and lower frequency limits at −6 dB, respectively, and *f_center_* is the center frequency of the transducer.

A customized wire phantom was prepared for measuring the axial and lateral resolutions of the transducer, which directly affect the transducer’s ability to sense microstructures for the FNA application. The phantom was composed of 90% de-ionized water, 5% n-propanol (to adjust for the speed of sound of approximately 1500 m/s [51]), and 5% porcine gelatin with a total volume of 500 mL. The ingredients were mixed with a stir bar over a stir plate and simultaneously heated to 40–45 °C. Meanwhile, two copper wires with a diameter of 160 μm were bonded in a customized mold with an axial separation of 500 μm and a lateral distance of 4 mm at an axial depth of 4 mm. Here, the axial distance corresponds to the distance from the transducer surface to the wire, while lateral distance is parallel to the direction of the transducer scanning motion. Once the liquid phantom cooled down to 30 °C, it was poured into the prepared custom mold and refrigerated for 24 h. After that, the 50 MHz transducer was bonded to a 3D motion stage with a step resolution of 100 μm. With a pulser/receiver (5900 PR, Olympus Corp, Waltham, MA, USA), the transducer was excited with a pulse of 2 μJ and a PRF of 200 Hz. For each test, 500 A-lines were collected with an oscilloscope (Agilent DSO7014B, Agilent Technologies Inc., Santa Clara, CA, USA) and the corresponding B-mode imaging was generated.

### 2.4. Tissue-Mimicking Phantom Preparation

We developed a melamine foam–gelatin phantom to mimic the acoustic contrast between healthy lung tissue and a lung nodule. The melamine foam material contained nanopores, and when submerged in a water tank, it became partially saturated. Pockets of air remained trapped inside the nanopores, mimicking the acoustic reflections of air-filled alveoli of lung tissue (mean volume: 4.2 × 10^6^ μm^3^) [52]. The gelatin, used to mimic a lung nodule, has a similar acoustic impedance to a human lymph node (approximately 1.64 MRayl) [53], allowing for acoustic wave propagation in the medium similar to that of the actual tissue.

For the gelatin preparation, degassed water was heated to 49 °C on a hot plate. A magnetic stir rod was used to stir the water at high speed while 10% m/V of type A, 300-bloom gelatin derived from acid-cured porcine skin (G2500, Sigma-Aldrich Corp., St. Louis, MO, USA) was slowly added. While stirring continuously at 49 °C, the solution was covered with aluminum foil to mitigate vapor loss to the surrounding air. Once the solution was transparent, it was removed from the hot plate and allowed to cool for 5 min. Subsequently, the solution was transferred to a mold with a height of 10 mm using a syringe, minimizing the introduction of air bubbles in the gelatin. We placed the mold in a refrigerator at 2 °C for 24 h to ensure the proper crosslinking of the solution ensued. Upon removal, the gelatin slab was extracted from the mold and a stencil was used to cut cylinders with a diameter of 8 mm and a height of 10 mm.

Melamine foam (Magic Eraser, Mr. Clean, Cincinnati, OH, USA) was cut into 4 equally sized pieces, each measuring 60 mm by 60 mm by 10 mm. A hole was cut in a corner of each foam sample and the gelatin cylinders were installed inside the holes.

### 2.5. Tissue-Mimicking Phantom Testing

**Figure 2:**
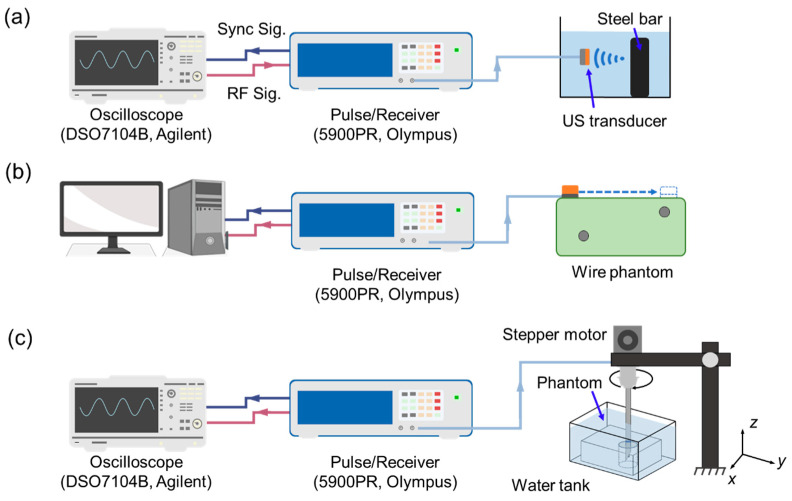
Experimental setup schematics for (**a**) miniaturized transducer characterization before mounting to the needle, (**b**) mechanical wire phantom scanning for resolution estimation, and (**c**) the needle rotation test for nodule/foam boundary detection.

To analyze the ability of a needle transducer to distinguish between nodules and healthy lung tissue, pulse/echo tests were conducted in the tissue-mimicking phantoms. A rotational scanning experiment was conducted to estimate the nodule boundary location. Figure 2c depicts the experimental setup for the phantom testing. The phantoms were placed in a tank with degassed water and the biopsy needle with an integrated ultrasound transducer was inserted from above using a 3D motion stage to control the vertical distance. Similar to the transducer characterization setup, a pulser/receiver and oscilloscope were used. The transducer was excited with a pulse energy of 2 µJ and a PRF of 200 Hz.

For each echo signal acquired with the transducer inside the gelatin, the time of flight, *t*, was recorded and the envelopes of the signals were analyzed. This was used along with the sound speed, *c*, in the 10% m/V gelatin (1520 m/s) to calculate the distance, *x*, from the transducer to the gelatin–foam boundary [50].

To acquire a cross-sectional image of the area surrounding the needle tip, a rotational scan was conducted in the phantom with the 50 MHz ultrasound needle as well as the 30 MHz ultrasound needle developed in our previous study [40]. For this test, the needle was inserted downward until the tip touched the face of the gelatin cylinder. Next, it was inserted 5 mm into the gelatin cylinder. Afterward, the echo signal from the gelatin–melamine foam interface was recorded. The needle was rotated in 3.6-degree increments, recording the echo signal between each displacement, for a total of 100 groups of A-line data for the full 360-degree rotation. The RF noise was acquired from the water tank without reflectors and was subtracted during data processing. To control the rotation, the needle was coupled to a stepper motor (Nema 17, STEPPERONLINE Inc., New York, NY, USA) using a 3D-printed linkage (KP3, KINGROON Tech Co., Ltd., Shenzhen, China) with PLA material (PLA 3D Printer Filament, HATCHBOX, Pomona, CA, USA). Subsequently, the stepper motor was connected to an Arduino UNO (Arduino Uno, Arduino, Ivrea, Italy) and stepper motor shield (Motor/Stepper/Servo Shield, Adafruit, New York, NY, USA). The Arduino and motor shield were then fixed inside 3D-printed housing to protect the circuitry during testing.

The RF signals acquired during the rotation test were compiled in MATLAB (MATLAB R2022a, The MathWorks, Natick, MA, USA), enveloped, and normalized with respect to each signal’s peak voltage. Using the aforementioned speed of sound in gelatin, the position of the signal corresponding to each time step was calculated. After generating B-mode images of the nodule cross-sections, the areas of the nodules were measured and compared to those measured from optical photographs. Additionally, eight radial lines were measured from the center of the nodule to its outer surface. These measured radii were spaced 45 degrees apart with respect to the nodule center.

## 3. Results

### 3.1. Characterization of the PMN-PT 1-3 Composite Material

**Figure 3:**
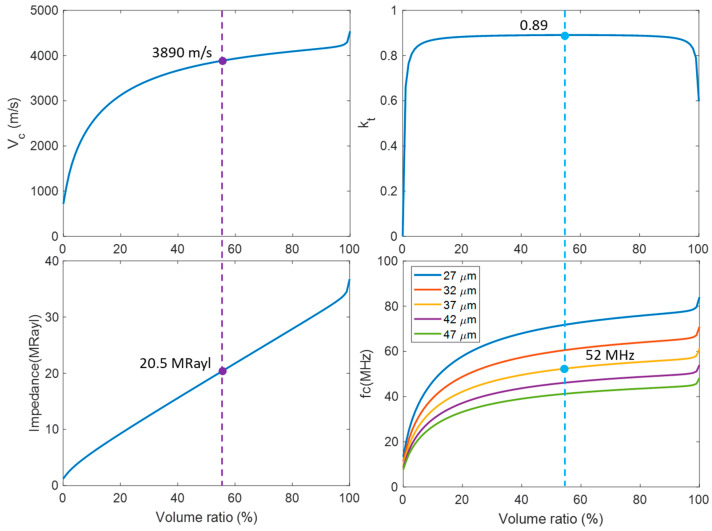
Variation in effective properties of 1-3 piezo-composite as a function of volume fractions.

**Figure 4:**
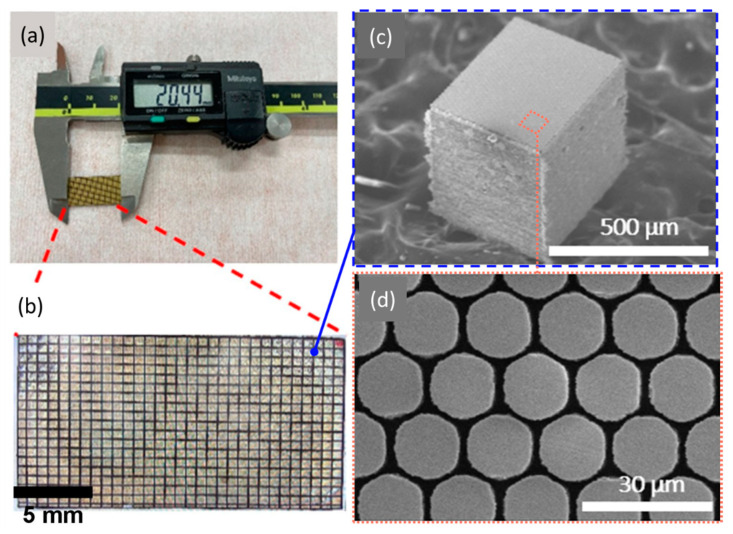
(**a**) Optical photograph of the prepared 1-3 composite material. (**b**) Optical photograph of the diced elements. (**c**) SEM images of one element of high-frequency composite with a size of 0.5 × 0.6 × 0.5 mm^3^. (**d**) Top view of honeycomb distribution PMN-PT pillars.

Figure 3 shows the desired properties and performance metrics of the 1-3 composite transducer with different volume ratios (V). Based on the simulation, at the volume ratio of 56%, the designed PMN-PT 1-3 composite had a high electromechanical coupling coefficient (*k_t_* = 0.89), relatively low acoustic impedance (Z = 20.5 MRayl), and a longitudinal velocity of 3890 m/s, corresponding to a thickness of 37 μm for the active layer thickness. Figure 4 shows the SEM images of the high-frequency composite with a pilar side wall angle of 86° to 88°, which was close to the desired vertical etched profile of 90° with a pillar height of 40 ± 5 μm. The resonant and anti-resonant frequencies of the material (*f_r_* and *f_a_*) were measured to be 52.0 MHz and 69.5 MHz, respectively. The effective electromechanical coupling coefficient *k_t_* [54] was calculated as

$${k_{t}}^{2}=\frac{\pi }{2}\cdot \frac{f_{s}}{f_{p}}\cdot \tan(\frac{\pi }{2}\cdot \frac{f_{p}-f_{s}}{f_{p}})$$

where *f_s_* and *f_p_* are series and parallel resonant frequencies, respectively, and the corresponding *k_t_* was measured to be 0.75 at a 56% volume ratio.

### 3.2. Characterization of the Miniaturized Transducer

**Figure 5:**
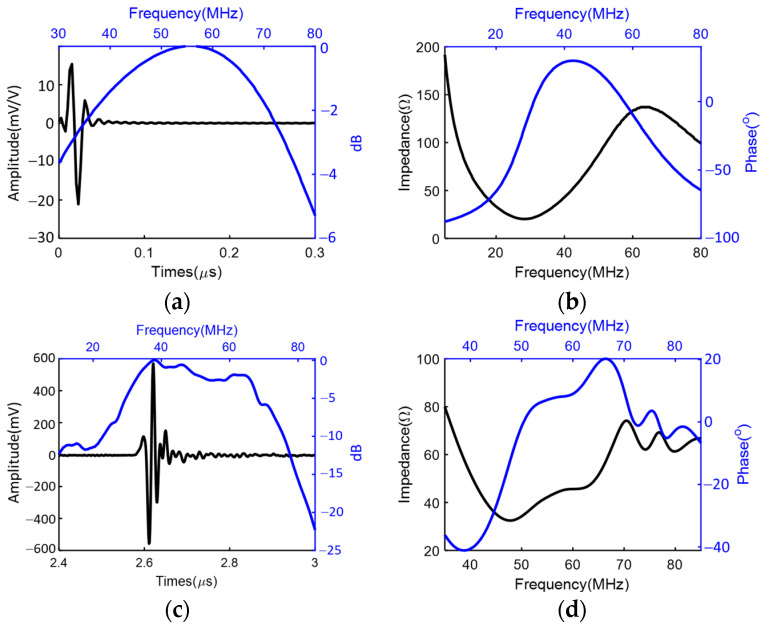
Pulse/echo response and electrical impedance of the HF single-element transducer: simulation results (**a**,**b**); measured results (**c**,**d**).

Figure 5a,b shows the simulated pulse/echo response and the electrical impedance of the high-frequency transducer. The simulated results showed a center frequency of 52.2 MHz with a −6 dB bandwidth of 110% and a loop sensitivity of −28.9 dB. At the center frequency, the simulated electrical impedance was estimated as 69 Ω. As a comparison, the measured pulse/echo test results showed a loop sensitivity of −28.4 dB with a center frequency of 49.8 MHz and a −6 dB bandwidth of 85.7%. The capacitance and dielectric loss at 1 kHz were measured as 445 pF and 11 mU, respectively. The measured results showed a minor deviation from the simulated results. With the high sensitivity and broad bandwidth, the prototyped transducer showed the capability to be used for high-resolution imaging and diagnosis.

### 3.3. Imaging Performance Characterization of the 50 MHz Transducer with Wire Phantom

**Figure 6:**
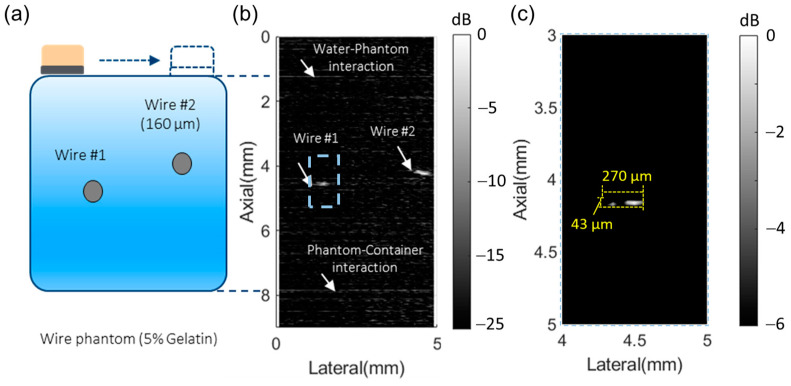
(**a**) Experiment setup for the wire phantom imaging. (**b**) Imaging for two wires using mechanical scanning with the 50 MHz transducer. (**c**) Axial and lateral resolution analysis with the HF transducer.

Figure 6a shows the schematic and relative position of the two wires in the phantom. Considering the attenuation of the phantom at high frequency (approximately 0.20 dB/cm/MHz [49]), the 50 MHz transducer showed high sensitivity with a signal-to-noise ratio (SNR) of 20 dB. Due to the high sensitivity of the transducer, the water–phantom and phantom–container interactions can be recognized at depths of 1 mm and 8 mm, respectively (Figure 6b). Figure 6c shows the enlarged imaging for the first wire with the 50 MHz transducer for the estimation of the axial and lateral resolution. The HF transducer showed a lateral resolution of 270 µm, which was most affected by the step resolution from the motion stage. Yet, the 50 MHz transducer showed an axial resolution of 43 μm, which indicated its potential for high accuracy in nodule boundary detection.

### 3.4. Tissue-Mimicking Phantom Results

**Figure 7:**
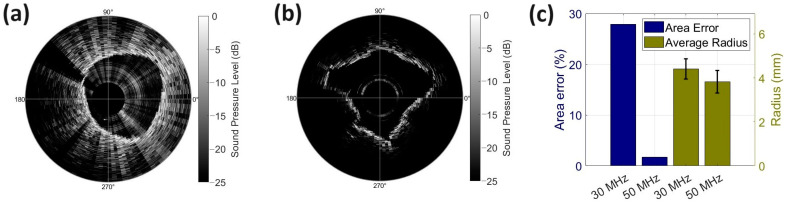
Grayscale plots of data acquired during rotation tests with (**a**) the 30 MHz ultrasound needle and (**b**) the 50 MHz ultrasound needle. (**c**) Comparison of area estimation error and average radius between the 30 MHz and 50 MHz transducers.

For the rotation study, the stepping angle and RF signals were used to produce polar grayscale plots. Figure 7a,b shows the measured sound pressure level during the rotation of the needles. The center of the plot is the location of the surface of the transducer. To limit the saturation effects of transducer ringdown on the received voltage, the first 2 μs were assigned 0 V of voltage for each RF signal. With 100 RF signals acquired at 3.6-degree intervals, a full cross-sectional image of the gelatin cylinder and surrounding melamine foam was constructed. Lighter segments on the plot correspond to higher sound pressure levels, while darker segments correspond to lower sound pressure levels. For both rotation experiments, in all four quadrants of the plot, the melamine foam–gelatin boundary is distinguished by areas of higher sound pressure. For the 30 MHz transducer, at the perimeter of this region and outward, scattered reflective segments continue to appear due to echoes from air pockets in the melamine foam. In the last quadrant of the 30 MHz rotation test, some large reflections were observed near the transducer surface. This was thought to be caused by twisting the needle, which sheared the gelatin, potentially allowing air to infiltrate the medium. Figure 7c demonstrates the error comparison for area and radii calculations for the 30 MHz and 50 MHz transducers. With an error of 1.74%, the 50 MHz transducer can generate an image with high accuracy compared to the 30 MHz transducer from our prior study (27.93%). Regardless, both ultrasound needles were capable of producing B-mode images of the gelatin and peripheral melamine sponge geometries, which demonstrates the potential for use with biopsy guidance.

## 4. Discussion

In this paper, we described the design, manufacturing, and characterization of a high-frequency ultrasound transducer for the FNA application in vitro. The miniaturized transducer was integrated with an 18-gauge needle and applied for mechanical scanning in the tissue-mimicking phantoms composed of gelatin and melamine foam. During the in vitro test, the needles were not only capable of estimating the distance to the gelatin–foam boundary but were also able to visualize structures with B-mode imaging, indicating the ability to improve the accuracy of the needle placement during FNA procedures.

To overcome the limited penetration depth for high-frequency ultrasound transducers, the performance of the device was improved by applying PMN-PT 1-3 composite. The newly designed 50 MHz transducer demonstrated a high loop sensitivity of −28.4 dB with a −6 dB bandwidth of 85.7%, which was 112% and 176% better compared to our previously reported 30 MHz PMN-PT single-crystal transducer [41]. Thus, the newly developed transducer reached a similar penetration depth (~9 mm) with better axial resolution (43 µm vs. 100 µm), which made the device suitable for minimally invasive biopsy needle applications.

With simplified control utilizing a stepper motor, we realized the mechanical scanning of the nodules for estimating the boundaries. Specifically, compared with the previously reported forward-looking high-frequency single-element ultrasound transducer, which focused on lumbar puncture guidance [55,56,57], our newly developed device provided abundant information for estimating the size of the nodule with the relative position between the needle and the lesion, which reduced the requirement for multiple biopsy needle operations. Furthermore, compared with electrical impedance measurement [8,17,34], the new device does not rely on frequency spectrum and error analysis, which simplifies data processing for real-time detection with A-lines shown in our previous work (PRF = 200 Hz) [41]. Once the biopsy needle is confirmed to be in the nodule, rotational B-mode imaging can be conducted for more detailed information about the nodule distributions with high resolutions. Although a low rotational speed was applied in this work due to safety concerns (10 RPM), a relatively higher frame rate may be realized with a more stable motor and automatic data acquisition system, which will be involved in our future works.

By rotating the needle, B-mode images were created, yielding a radial scanning resolution of 3.6 degrees, corresponding to a lateral resolution of 470 µm at an axial depth of 7.6 mm. With a dynamic range of 25 dB, the geometries of the gelatin cylinder and outlying air pockets were observed. When comparing the B-mode images, the 50 MHz and 30 MHz transducers generated the boundary with estimated axial resolutions of 43 µm and 100 μm, respectively, with corresponding maximum contrast-to-noise ratios of 32.64 dB and 29.01 dB. Thus, a clearer gelatin–melamine foam boundary was demonstrated with the 50 MHz transducer. It is important to highlight that the gelatin cylinders conformed to the shape of the melamine foam upon insertion, establishing an arbitrarily shaped boundary between the gelatin and saturated melamine foam. Hence, in the generated B-mode images, the gelatin boundary does not appear circular. After being embedded in the melamine foam, the designed nodule tested with the 50 MHz transducer had a radius of 3.82 ± 0.52 mm versus 4.41 ± 0.47 mm for the nodule tested with the 30 MHz transducer. These true radius values were measured at 45-degree increments about the center of the nodule using optical photographs. Even with larger nodule radius variation, B-mode imaging from the 50 MHz transducer showed less error for the nodule area estimation compared with the 30 MHz transducer (1.74% vs. 27.93%). The enhanced accuracy in estimating nodule area is a result of improved bandwidth and loop sensitivities in the 50 MHz transducer. The higher −6 dB bandwidth of the 1-3 composite transducer (31% vs. 85.7%) contributes to enhanced axial resolution from 100 μm to 43 μm. Increased loop sensitivity (112%) leads to a larger contrast-to-noise ratio from 29.01 dB to 32.64 dB. Additionally, the smaller size of the newly developed 50 MHz transducer allowed for improved alignment with the needle tip during fabrication, reducing the tilting angle during imaging. These combined improvements facilitate clearer imaging of boundaries between adjacent tissues, enabling a more precise area estimation with the newly developed 50 MHz transducer. This evidence suggests that the device is capable of estimating the nodule size, and that the device may also be useful for calculating the growth of a lesion over time, which could be helpful for monitoring disease progression, or response to treatment.

With promising results, there are still several limitations to this work. Although the foam–gelatin phantoms have been applied for mimicking lung nodules and surrounding tissues for in vitro testing, further ex vivo and in vivo testing will be required to include pulmonary alveoli and blood vessels in inflated lung tissues. Additionally, due to the relatively large rotational step (3.6 degrees/step), the lateral resolution was limited, and the nodule size was overestimated. For future studies, a 0.9-degree stepping angle will be considered to improve the annular resolution of the peripheral lung features to approximately 120 μm, while keeping the frame rate high enough for real-time detection. To further improve the performance of the device, it could be beneficial to integrate both insertion and rotational mechanical scanning techniques to give the clinical operator increased visibility and positional awareness of the needle tip.

## 5. Conclusions

This investigation successfully demonstrated the feasibility of guiding a fine-needle biopsy using an integrated ultrasound transducer in vitro. With micromachined PMN-PT 1-3 composite, a miniaturized transducer (0.6 × 0.5 × 0.5 mm^3^) was designed, fabricated, and characterized with high loop sensitivity (−28.4 dB) and broad −6 dB bandwidth (87%). For in vitro testing, the transducer was controlled by a stepper motor and 3D motion stage to perform mechanical scanning of a nodule within a phantom. This integrated transducer demonstrated the ability to achieve high-resolution imaging with an axial resolution of <100 μm for discerning nodule edges. Moreover, it exhibited an error of 1.74% in estimating nodule size compared to actual dimensions. These results show the promising potential of biopsy-needle-integrated transducers in enhancing the accuracy of EUS-FNA for clinical applications.

## Figures and Tables

**Table 1:** Axial and lateral resolutions for devices previously developed for FNA needle integration.

Sensing Modality	Axial Resolution	Lateral Resolution	Reference
Electrical Impedance	N/A	200 μm	Li, T. et al. [17,18]
US Array	33.2 μm	115.6 μm	Cummins, T. et al. [18]
US Array	90 μm	392 μm	Jung, H. et al. [19]
Single Element US Transducer	53 μm	400 μm	Huang, C. et al. [38,39]

**Table 2:** Design parameters for the single-element 50 MHz 1-3 composite transducer.

	Material	Thickness	Velocity	Density	Acoustic Impedance
Active layer	PMN-PT 1-3 composite	37 μm	3890 m/s	5272 kg/m^3^	20.5 MRayl
Matching layer	Parylene C	9 μm	2770 m/s	1140 kg/m^3^	3.16 MRayl
Backing layer	E-solder 3022	500 μm	2110 m/s	2590 kg/m^3^	5.5 MRayl

## Data Availability

Data is available upon request.
